# Frailty predicts adverse clinical outcomes in patients with moderate to severe chronic kidney disease

**DOI:** 10.18632/aging.206239

**Published:** 2025-04-15

**Authors:** Chiung-Ying Huang, Hsiao-Mei Tsao, Shu-Ling Liang, Tai-Shuan Lai, Yung-Ming Chen

**Affiliations:** 1 Department of Medicine, National Taiwan University Hospital, Bei-Hu Branch, Taipei, Taiwan; 2 Department of Internal Medicine, Division of Nephrology, National Taiwan University Hospital, Taipei, Taiwan; 3 Department of Internal Medicine, College of Medicine, National Taiwan University, Taipei, Taiwan

**Keywords:** frailty, Fried’s frailty phenotype, SOF index, frailty index, chronic kidney disease

## Abstract

Background and Aim: Frailty predicts adverse clinical outcomes in older adults. Its prognoses in individuals with specific illnesses have not been fully explored. This study aimed to investigate the impact of frailty by using a semiautomated instrument in patients with advanced chronic kidney disease (CKD).

Methods and Results: In this prospective study, patients with CKD3b-5 before dialysis and aged ≥55 years with a clinical frailty scale of ≤5 were enrolled. Frailty was assessed by three commonly-used evaluation tools, i.e., Fried’s frailty phenotype, Study of Osteoporotic Fractures (SOF) index, and Frailty index of 80 risk variables (FI80) incorporated in a semiautomated platform. Logistic regression, Kaplan–Meier analysis, and Cox proportional hazards models were used to analyze the predictors for frailty and the impact of frailty on composite outcomes of dialysis and overall death.

Among 315 patients, the mean age was 73.1 years, and the estimated glomerular filtration rate was 22.2 ml/min/1.73 m^2^. The prevalence of frailty was 6.2% by Fried’s frailty phenotype, 0.6% by SOF index, and 26.7% by FI80. Logistic regression analysis showed that age, but not CKD severity or proteinuria, was the most consistent predictor for frailty across the three evaluative tools. During an average follow-up period of 1.7 years, the incidences of kidney failure resulting in dialysis, overall death, or hospital admission were 10.5, 0.6, and 15.2 per 1,000 patient-month, respectively. Kaplan-Meier analysis revealed that frail patients identified by FI80 exhibited worse composite outcomes than their prefrail and robust counterparts (log-rank test, *P* = 0.01). Multivariate Cox models confirmed that frailty defined by FI80 predicted adverse composite outcomes (HR 3.51, 95% CI: 1.20, 10.22).

Conclusions: Frailty is common among CKD patients, and its prevalence increases with age and disease advancement. The frailty status identified by the FI80 effectively predicted end-stage kidney disease or death in patients with advanced CKD.

## INTRODUCTION

Frailty is an age-related condition stemming from a diverse array of factors. It is characterized by a progressive decline across multiple organ systems and a depletion of physiological reserves. Frailty extends beyond physical limitations and encompasses cognitive, social, and psychological decline [1, 2], leading to reduced activity and increased vulnerability to stressors [3]. The most commonly used criteria to define frailty are the phenotypic model proposed by Fried et al. [4], which includes the following five components: weakness, slowness, low activity, exhaustion, and weight loss. Another simplified phenotypic version is the Study of Osteoporotic Fractures (SOF) index, comprising three components: weight loss, inability to rise from a chair five times, and a reduced energy level. Both models are well-validated frailty evaluations for community-dwelling older adults [5, 6]. The weight loss criteria may be problematic in patients with advanced chronic kidney disease (CKD) because of fluctuations in fluid status.

In contrast to the phenotypic models that focus mainly on assessing physical domains, the deficit cumulative model proposed by Kenwood et al. [7] encompasses physical, cognitive and socioeconomic factors, when constructing a frailty index (FI). The effect of frailty index on patient’s clinical outcomes has been examined in many studies under several settings, including patients with CKD, hip fracture, malignancy, HIV infection and community-dwelling older people [8–15]. While this FI model may provide a more comprehensive evaluation of frailty status than Fried’s model or the SOF index, the time-consuming and labor-intensive nature of the assessment hinders its broader application. The Pictorial Clinical Frailty Scale is a more concise tool derived from the concept of FI for frailty evaluation; however, its interpretation is judgement-based, requiring experienced physicians to maintain inter-rater reliability.

More recently, we reported the utility of a novel, electronic FI of 80 risk variables (FI80), which also integrates various elements of Fried’s frailty phenotype and the SOF index, in predicting healthcare outcomes among non-frail and pre-frail older adults in the community [16].

Frailty is highly prevalent in individuals with CKD across all stages and is associated with adverse clinical outcomes [17]. The estimates of frailty range from 7% in community-dwelling patients with mild CKD, 19% in a mixed CKD population, to 42.6% in patients with severe CKD, and 53.8% in a predialysis population [8, 17–19]. Although frailty has proposed as a significant contributor to morbidity and mortality in patients with kidney diseases [8, 20–22], integrating this concept into the care of nephrology patients remains a challenge, partly because of the lack of a useful tool to define frailty status as well as uncertainties over the evolution of this syndrome on clinical outcomes. Therefore, because of the high incidence and prevalence of end-stage kidney disease (ESKD) in Taiwan [16, 17], we conducted a prospective study recruiting patients with moderate-to-severe CKD (Stages 3b to 5). We investigated the prevalence of frailty using a semi-automated FI80 platform [16, 23] which also yielded the output of the Fried frailty phenotype and SOF index during the same round of assessment. Predictors of frailty were analyzed, and the predictive abilities of three frailty assessment methods in relation to clinical outcomes were clarified.

## METHODS

### Participants

We conducted a prospective cohort study of 315 nephrology outpatients in a tertiary hospital in Taiwan from June 1, 2019, to Dec 31, 2020. There are 53 patients censored due to lost-to follow up and no patient censored due to kidney transplant. Patients more than 55-years-old, with eGFR less than 45 mL/min/1.73 m^2^, and clinical frailty scale ≤5 were included. We excluded patients who had received renal replacement therapy, had a pacemaker or metal implant, had an active malignancy, or were diagnosed with psychological disorders. Patient characteristics, including age, sex, height, weight, body mass index, comorbidities, and laboratory data (serum albumin, serum potassium, eGFR, serum phosphorus, blood urea nitrogen, serum creatinine, hemoglobin, white blood cell count, and urine protein-to-creatinine ratio (UPCR)) were collected to form an electronic medical chart. Follow-ups for each individual were continued until dialysis, death or at the end of December 2021.

### Measurements

Frailty assessments at entry and at 6 months were performed under the guidance of a trained assistant. The assessment used a semiautomated BabyBot vital data recording system (Netown Corporation, Taipei, Taiwan), which incorporated components for three frailty-assessing instruments, i.e., the Fried frailty phenotype, SOF index, and deficit-accumulation FI80 [4, 7, 24–26].

#### Fried frailty phenotype

The Fried phenotype includes five components: weakness (grip strength), slow walking speed, low physical activity, self-reported exhaustion, and unintentional weight loss. The score range is 0–5, the higher the score indicates heavier frailty, 0 (namely robust), 1–2 into prefrailty, and ≥3 into frailty.

#### SOF index

SOF frailty includes three components: unintentional weight loss, the inability to rise from a chair five times without using the arms, and reduced energy levels. The score range is 0–3, the higher the score indicates heavier frailty, 0 (namely robust), 1 into prefrailty, and ≥2 into frailty.

#### Deficit-accumulation FI80

Each individual’s frailty risk was evaluated using the FI80, which encompasses a 68-item set of self-reporting questionnaires spanning various domains including: cognition and mood, comorbidity, nutrition and physiology, fall risk, activity, and communication. The questionnaires were administered using a touchscreen tablet. The evaluation also included 12 objective measurements, including a 3-in-1 machine (OMRON Automatic Blood Pressure Monitor; BabyBot Pulse Oximeter) for vital signs, a bioelectrical impedance analyzer (Tanita BC-418, Tokyo, Japan) for body composition and body mass index, a hand-held dynamometer with digital output for hand grip strength, gait speedometers equipped with infrared sensors to gauge walking speed, and a cushion-type pressure sensor for the timed Up and Go test and 5 times sit-to-stand test. The FI system assigned equal weights to all 80 included items. The threshold for defining frailty in this study was established utilizing the methodology by Rockwood et al. [25, 26]. Scores below 0.11 indicated robust health, scores ranging from 0.11 to 0.21 were classified as prefrailty, and scores exceeding 0.21 indicated frailty.

### Statistical analysis

All variables are reported as mean ± SD (or with 95% confidence intervals where appropriate) for continuous variables and as frequencies or percentages for categorical variables. ANOVA was used for analysis between groups where appropriate. Differences in frequency were tested using Chi-square analysis. Logistic regression was used to analyze the association between predictors and frailty status defined by Fried, SOF, and FI80. Kaplan–Meier survival analysis and Cox regression proportional hazard analysis were used to analyze survival rates between groups and predictors of survival, respectively. Statistical significance was set at *P* < 0.05. Statistical analyses were performed using SPSS 19.0 for Windows (SPSS Inc., IL, USA).

## RESULTS

**Figure 1:**
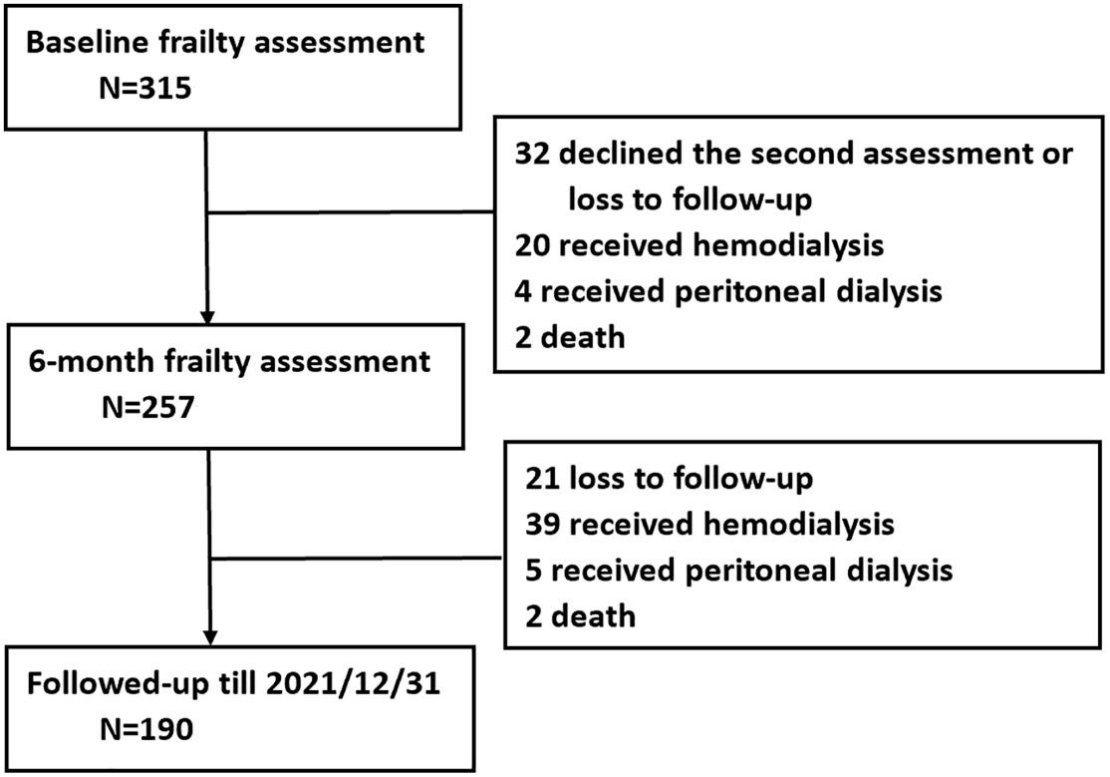
Flowchart of the study.

A total of 315 patients (mean age 73.1 years, female 36.2%, eGFR 22.2 ml/min/1.73 m^2^) were recruited in this study (Figure 1). The baseline demographic, clinical, and laboratory values are shown in Table 1. Patients were categorized into three groups (robust, pre-frailty and frailty) according to three frailty measurements (Fried’s phenotype, SOF index and FI80).

**Table 1:** Baseline demographics and laboratory data of the participants.

	**Overall *n* = 315**	**Stage 3b *n* = 83**	**Stage 4 *n* = 145**	**Stage 5 *n* = 87**	***P***
	**Mean ± SD**	**Mean ± SD**	**Mean ± SD**	**Mean ± SD**	
**Demographics**					
Age, years	73.1 ± 9.1	73.8 ± 9.2	73.1 ± 9.0	72.3 ± 9.3	0.591
**Sex**					
Male	201 (63.8)	64 (77.1)	91 (62.8)	46 (52.9)	0.004^*^
Female	114 (36.2)	19 (22.9)	54 (37.2)	41 (47.1)	
**Laboratory**					
eGFR, mL/min/1.73 m^2^	22.2 ± 10.2	35.5 ± 4.4	21.8 ± 4.4	10.2 ± 3.0	<.001^*^
Albumin, g/dL	4.0 ± 0.5	4.1 ± 0.6	4.0 ± 0.4	4.0 ± 0.4	0.315
BUN, mg/dL	51.4 ± 22.3	33.6 ± 10.0	47.4 ± 13.9	75.1 ± 22.2	<.001^*^
WBC, k/μL	7.0 ± 2.3	6.9 ± 2.2	7.0 ± 2.0	7.3 ± 2.7	0.378
Hb, g/dL	10.8 ± 1.9	12.0 ± 1.7	10.9 ± 1.7	9.5 ± 1.5	<.001^*^
K, mmol/L	4.7 ± 0.6	4.6 ± 0.5	4.8 ± 0.6	4.8 ± 0.7	0.033^*^
Phosphate, mg/dL	4.1 ± 0.9	3.5 ± 0.5	3.9 ± 0.6	4.9 ± 1.0	<.001^*^
UPCR, mg/g	2098.6 ± 2514.7	1186.3 ± 2097.0	1927.1 ± 2379.6	3206.9 ± 2691.8	<.001^*^

^*^P < 0.05, using Student’s *t*-test for continuous variables or the chi-square test for categorical variables. Abbreviations: eGFR: estimated glomerular filtration rate; BUN: blood urea nitrogen; WBC: white blood cell; Hb: hemoglobin; K: potassium; UPCR: urine protein to creatinine ratio.

**Figure 2:**
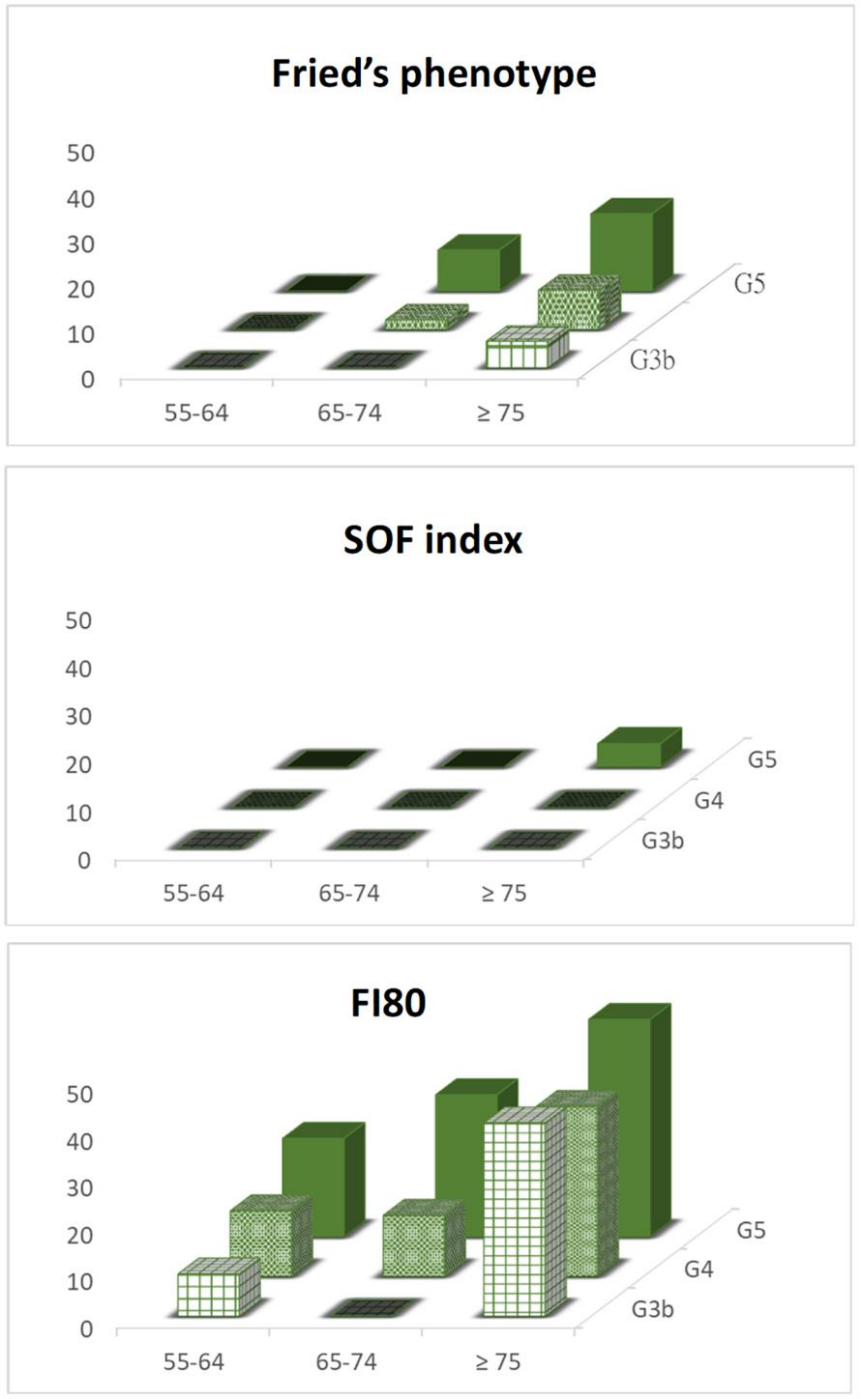
The prevalence of frailty status assessed by different measurements and stratified by age and CKD stages.

The overall prevalence of frailty was 6.2% by Fried’s frailty phenotype, 0.6% by SOF index, and 26.7% by FI80. Patients with Stage 5 CKD and those over the age of 75 years displayed the highest prevalence of frailty: 10.8% and 10.4% by Fried’s frailty phenotype, 2.2% and 1.4% by SOF index, 35.5% and 40.3% by FI80, respectively (Figure 2). Further analysis using logistic regression revealed that age was the most consistent predictor of frailty across the three different measurements, more so than CKD severity or proteinuria, which were significant only in the FI80 model (Table 2). Other predictors included anemia and high BUN levels in the Fried phenotype model and high UPCR and BUN levels in the FI80 model. Over the 6-month follow-up, patients with proteinuria tended to fare the worst in frailty evolution by FI80 (worse, *n* = 28/29, 96.5% by FI80, *x*^2^ test, *P* = 0.051), compared with patients without proteinuria (Table 3).

**Table 2:** Predictors of frailty defined by (A) Fried’s phenotype, (B) SOF index, and (C) FI80.

**Characteristics**	**Univariate analysis**			**Multivariate analysis**		
	**ORs**	**95% (C.I.)**	***P***	**ORs**	**95% (C.I.)**	***P***
**(A) Fried’s phenotype (*N* = 306)**						
Male sex	0.904	(0.575, 1.423)	0.6638			
Age	1.094	(1.065, 1.124)	<.0001^*^	1.098	(1.07, 1.13)	<.0001^*^
CKD stage						
3	Reference					
4	0.886	(0.520, 1.508)	0.6556			
5	1.25	(0.690, 2.265)	0.4621			
Laboratory						
eGFR, mL/min/1.73 m^2^	0.988	(0.967, 1.01)	0.28			
UPCR, g/g	1	(1, 1)	0.7219			
albumin, g/dL	0.617	(0.386, 0.987)	0.0441^*^	0.953	(0.585, 1.554)	0.8163
Hb, g/dL	0.763	(0.676, 0.861)	<.0001^*^	0.8	(0.695, 0.920)	0.0018^*^
BUN	1.013	(1.003, 1.024)	0.0092^*^	1.013	(1.001, 1.024)	0.0361^*^
K, mmol/L	1.032	(0.720, 1.478)	0.864			
Phosphate, mg/dL	1.147	(0.888, 1.481)	0.2928			
**(B) SOF index (*N* = 306)**						
Male sex	1.665	(0.919, 3.017)	0.0928			
Age	1.109	(1.068, 1.153)	<.0001^*^	1.109	(1.068, 1.153)	<.0001^*^
CKD stage						
3	Reference					
4	0.893	(0.428, 1.863)	0.7631			
5	1.322	(0.609, 2.868)	0.4803			
Laboratory						
eGFR, mL/min/1.73 m^2^	0.994	(0.966, 1.023)	0.694			
UPCR, g/g	1	(1, 1)	0.6779			
Albumin, g/dL	0.97	(0.523, 1.799)	0.9235			
Hb, g/dL	0.886	(0.754, 1.04)	0.138			
BUN	1.004	(0.991, 1.017)	0.554			
K, mmol/L	0.84	(0.515, 1.370)	0.485			
Phosphate, mg/dL	1.222	(0.880, 1.696)	0.2316			
**(C) FI80 (*N* = 306)**						
Male sex	1.536	(0.966, 2.444)	0.0698			
Age	1.072	(1.045, 1.101)	<.0001^*^	1.094	(1.062, 1.128)	<.0001^*^
CKD stage						
3	Reference					
4	1.182	(0.684, 2.042)	0.5493			
5	1.857	(1.011, 3.412)	0.0461^*^			
Laboratory						
eGFR, mL/min/1.73 m^2^	0.976	(0.954, 0.997)	0.0281^*^	1.028	(0.991, 1.066)	0.1460
UPCR, g/g	1	(1, 1)	0.0028^*^	1	(1, 1)	0.0018^*^
Albumin, g/dL	0.614	(0.382, 0.989)	0.0451^*^	1.176	(0.664, 2.081)	0.5789
Hb, g/dL	0.867	(0.769, 0.977)	0.0192^*^	0.968	(0.833, 1.125)	0.6746
BUN	1.013	(1.003, 1.024)	0.0092^*^	1.022	(1.004, 1.040)	0.0145^*^
K, mmol/L	0.964	(0.668, 1.392)	0.8449			
Phosphate, mg/dL	1.365	(1.051, 1.772)	0.0195^*^	1.155	(0.782, 1.705)	0.4696

^*^P < 0.05. Abbreviations: CKD: chronic kidney disease; eGFR: estimated glomerular filtration rate; UPCR: urine protein-to-creatinine ratio; Hb: hemoglobin; K: potassium; BUN: blood urea nitrogen.

**Table 3:** Demographics, CKD stages, and proteinuria magnitude between different patterns of frailty evolution over 6 months.

	**(A) Fried’s phenotype (*N* = 264)**			**(B) SOF index (*N* = 260)**			**(C) FI80 (*N* = 264)**		
	**Worse (*n* = 55)**	**Stable/better (*n* = 209)**	***P***	**Worse (*n* = 22)**	**Stable/better (*n* = 239)**	***P***	**Worse (*n* = 41)**	**Stable/better (*n* = 223)**	***P***
Male sex	26 (47.27)	147 (70.33)	0.001^*^	13 (59.09)	159 (66.81)	0.455	25 (60.98)	148 (66.37)	0.504
Age, years, *n*, %			0.313			0.51			0.146
55–64	8 (14.55)	44 (21.05)		5 (22.73)	47 (19.75)		12 (29.27)	40 (17.94)	
65–74	19 (34.55)	81 (38.76)		6 (27.27)	94 (39.5)		11 (26.83)	89 (39.91)	
≥75	28 (50.91	84 (40.19)		11 (50)	97 (40.76)		18 (43.90)	94 (42.15)	
CKD stages, *n*, %			0.068			0.068	55–64		0.602
3b	12 (21.82)	63 (30.14)		6 (27.27)	69 (28.99)		9 (21.95)	66 (29.6)	
4	22 (40)	98 (46.89)		6 (27.27)	112 (47.06)		20 (48.78)	100 (44.84)	
5	21 (38.18)	48 (22.97)		10 (45.45)	57 (23.95)		12 (29.27)	57 (25.56)	
Proteinuria, *n*, %			0.744			0.262			0.051
Negative	6 (13.95)	24 (16)		4 (25)	12 (75)		1 (3.45)	29 (17.68)	
Positive	37 (86.05)	126 (84)		25 (14.45)	148 (85.55)		28 (96.55)	135 (82.32)	

^*^P < 0.05. Abbreviation: CKD: chronic kidney disease.

**Figure 3:**
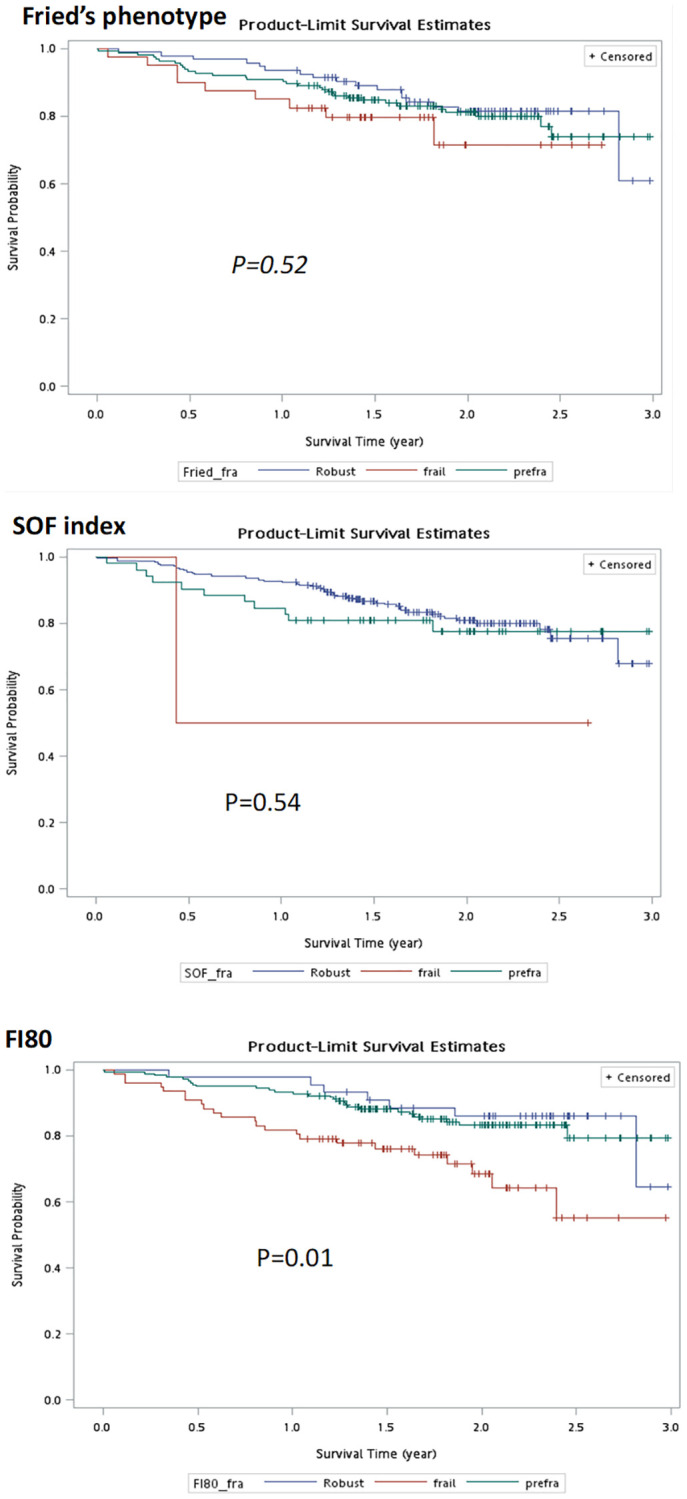
Kaplan–Meier curves of frailty status defined by different measurements for composite outcomes.

During an average follow-up period of 1.7 years, there were 68 (21.6%) kidney failures required long-term dialysis, 4 (1.3%) died from any cause, and 98 (31.1%) hospital admissions. The Kaplan–Meier plots for composite outcomes (chronic dialysis or overall death) stratified by the three frailty measurements are depicted in Figure 3. The frail group identified by FI80, but not by Fried’s scale or the SOF index, displayed significantly worse composite outcomes than their prefrail and robust counterparts (*P* = 0.01). Consistent with this finding, the unadjusted Cox regression analysis found HRs and confidence intervals of frailty for composite outcomes, using the robust group as reference, were 1.5 (0.7, 3.1) by Fried’s phenotype, 2.8 (0.4, 20.2) by SOF index and 2.5 (1.1, 5.5) by FI80, respectively. After adjusting for age, sex, and proteinuria, frailty as defined by the SOF index and FI80 predicted composite outcomes (HRs, SOF index 10.3 (1.3, 81.5), FI80 3.5 (1.2, 10.2), respectively). Because the SOF index does not contain comorbidities relevant to CKD, the SOF model was further adjusted for diabetes, and cardiovascular disease. After correction, the risk of adverse outcomes posed by the SOF-frailty was substantially attenuated (7.39 (0.93, 58.78)), and diabetes stood out as an independent predictor (2.16 (1.22, 3.82)), along with existing proteinuria (9.59 (1.31, 69.82)) for composite outcomes (Table 4). The proportional-hazards assumption with the Schoenfeld residuals test in the association of FI80 with clinical outcomes was also assessed. The test is not statistically significant for each of the covariates (FI80, age, sex, Proteinuria), and the global test is also not statistically significant (*p* = 0.718). The proportional hazards (PH) assumption is satisfied (Supplementary Figure 1).

**Table 4:** Multivariate Cox proportional hazards models showing predictors of composite outcomes (dialysis or death).

	**Model 1: Fried’s phenotype**		**Model 2: SOF**		**Model 3: FI80**	
	**HRs**	**(95% C.I.)**	**HRs**	**(95% C.I.)**	**HRs**	**(95% C.I.)**
**Variables**						
Robust	1.00	(reference)	1.00	(reference)	1.00	(reference)
Pre-frailty	1.34	(0.73, 2.44)	1.93	(0.99, 3.76)	1.75	(0.62, 4.95)
Frailty	1.60	(0.69, 3.67)	7.39	(0.93, 58.78)	3.51^*^	(1.20, 10.22)
**Age group, y**						
55–64	1.00	(reference)	1.00	(reference)	1.00	(reference)
65–74	0.65	(0.34, 1.29)	0.66	(0.34, 1.30)	0.71	(0.36, 1.38)
≥75	0.63	(0.32, 1.23)	0.58	(0.30, 1.15)	0.57	(0.30, 1.09)
Sex female	1.15	(0.69, 1.93)	1.10	(0.65, 1.85)	0.99	(0.59, 1.67)
**Proteinuria**						
Negative	1.00	(reference)	1.00	(reference)	1.00	(reference)
Positive	9.29^*^	(1.27, 67.67)	9.59^*^	(1.31, 69.82)	10.95^*^	(1.51, 79.39)
**DM**						
No	1.00	(reference)	1.00	(reference)		
Yes	2.03^*^	(1.16, 3.57)	2.16^*^	(1.22, 3.82)		
**CVD**						
No	1.00	(reference)	1.00	(reference)		
Yes	0.79	(0.46, 1.38)	0.82	(0.47, 1.41)		

The three models were first adjusted for age, sex, and proteinuria. Models 1 and 2 were further adjusted for diabetes, hypertension, and cardiovascular disease. This adjustment was not done in Model 3 owing to the inclusion of these variables within FI80. ^*^P < 0.05. Abbreviations: DM: diabetes mellitus; CVD: cardiovascular disease.

## DISCUSSION

In this prospective study, we demonstrated that a novel frailty-assessing measurement based on the deficit accumulation model, FI80 [16, 23] effectively predicted composite outcomes of chronic dialysis or overall death in patients with CKD stages 3b–5 prior to ESKD. These findings underscore the importance of monitoring frailty, besides the traditional proteinuria and diabetes mellitus. Assessment of frailty can be time and manpower-intensive. The FI80 encompasses 80-item deficits covering the physical, cognitive, social, and psychological domains of frailty. It has been employed to trace frailty evolution and predict clinical outcomes in non-frail or prefrail community-dwelling older adults and ESKD patients undergoing chronic peritoneal dialysis [16, 23].

Patients with CKD are known to exhibit an increased predisposition to frailty [22]. The prevalence of frailty is estimated 14–43% in non-dialysis-dependent CKD patients and 30–82% in dialysis individuals, depending on the methods used [27–30]. In the present study, the prevalence of frailty was 26.7% by FI80, which was higher than that determined by Fried frailty phenotype (6.2%) and SOF index (0.6%). The large variation in the prevalence based on different measurements could be related to the number of parameters embedded within each tool. The FI80 contains 80 items (12 objective measurements and 68 self-reported questionnaires), whereas Fried’s scale and the SOF index comprise five and three items, respectively.

As mentioned above, the prevalence of frailty defined by the FI80 was more than four times higher than that determined by the Fried frailty phenotype. This is concordant with the results found in older community members, which showed that many people classified as phenotype-robust had high FI values and few people with phenotypic frailty had low FI values [26, 27]. Among patients with ESKD and advanced CKD, cumulative deficits in FI indicated a higher prevalence of frailty than phenotype-based measurements [18, 31, 32].

A plethora of predictors for frailty have been identified in patients with kidney disease, with or without chronic dialysis, which included old age, female, advanced CKD stage, hypoalbuminemia, and anemia [30, 33, 34]. In this study, old age was the most potent predictor of frailty irrespective of the measurements used [35, 36]. In addition, CKD severity (Stage 5 vs. 3b), presence of proteinuria, eGFR, and serum phosphate levels were also associated with frailty defined by FI80, but not Fried’s scale or SOF index. The latter findings highlight the uniqueness of the FI80 as the most sensitive tool for frailty screening, given that no laboratory parameters are included in the instrument. Traditional factors such as age, BUN and hemoglobin were found to be either predictors or protectors of frailty across different measurements.

Among the 264 participants who underwent a second frailty assessment at 6 months, 20.8% of them became frailer by Fried’s scale, with 47.2% of them being male. This finding coincides in part with the “sex-frailty paradox,” which emphasizes that females are inclined to develop frailty despite having a lower risk of mortality across all ages [30, 37]. It is worth mentioning that the presence of proteinuria tended to be associated with worsening frailty, as defined by FI80, but not by Fried’s scale or the SOF index. This suggests a potential connection between proteinuria severity and frailty progression [38, 39], which warrants further investigation.

During the follow-up, we observed 21.6% of patients commenced long-term dialysis, which was 17-fold higher than overall death (1.3%). This coincides with our previous observations that Taiwanese and perhaps Asian patients with CKD are more likely to develop ESKD than those who died of any cause, compared with their Western counterparts [40]. In addition to proteinuria and diabetes, we identified frailty as an independent predictor of chronic dialysis and overall death. These observations are reminiscent of a previous report that showed that a higher severity of frailty (as defined by the Clinical Frailty Scale) at dialysis initiation was associated with higher mortality in incident chronic dialysis patients [41]. Nonetheless, our study is the first to show such an association in patients with advanced CKD before dialysis. Since the initiation of chronic dialysis was more likely to occur than overall death, we surmise that frailty might be viewed as a surrogate marker of ESKD development, thus prompting the need for concomitant frailty management during multidisciplinary care for CKD [42].

The strength of this study is that we reported a semiautomated instrument for frailty assessment, FI80, which predicted adverse outcomes more effectively than Fried’s frailty phenotype and the SOF index in patients with advanced CKD. This finding is consistent with our recent report on non-frail to pre-frail community-dwelling older adults [16, 43]. However, the FI80 instrument is capable of yielding three different frailty scores (FI, Fried’s scale, and SOF index) during the same round of assessment, so there is no need to argue which measurements performed better as long as the results are complementary to each other and applied to patient care. This study had some limitations. First, this was a single-center study, and the patients were recruited from a tertiary hospital. Therefore, patients may have multiple illnesses that are not representative of all patients with advanced CKD. Second, the self-reported questionnaires displayed on the touchscreen tablet interface might have been subject to reporting bias. However, with the assistance of trained personnel, we showed that evaluation with the FI80 platform could save time in terms of the manpower required. Any inconsistency during evaluation could thus be controlled to a negligible level [16]. Third, the weight loss criterion may be problematic in advanced CKD populations because of fluctuating fluid status. Nevertheless, the weighting of this factor was diluted by the remaining 79 deficits built in the FI80 measurement.

## CONCLUSIONS

Frailty is common among non-dialysis CKD patients, and its prevalence increases with age and the advancing stages of the disease. The frailty status identified by using FI80 incorporated in a semiautomated platform effectively predicted ESKD or death in patients with moderate-to-severe CKD.

## Supplementary Materials

Supplementary Figure 1
