# Supplementary material for: Frailty predicts adverse clinical outcomes in patients with moderate to severe chronic kidney disease
Source: Aging (Albany NY). 2025 Apr 15;17(4):1060–72. doi: 10.18632/aging.206239 (PMC12074821; doi:10.18632/aging.206239)
Supplement: Supplementary Figure 1 [file aging-17-206239-s001.pdf]

SUPPLEMENTARY FIGURE

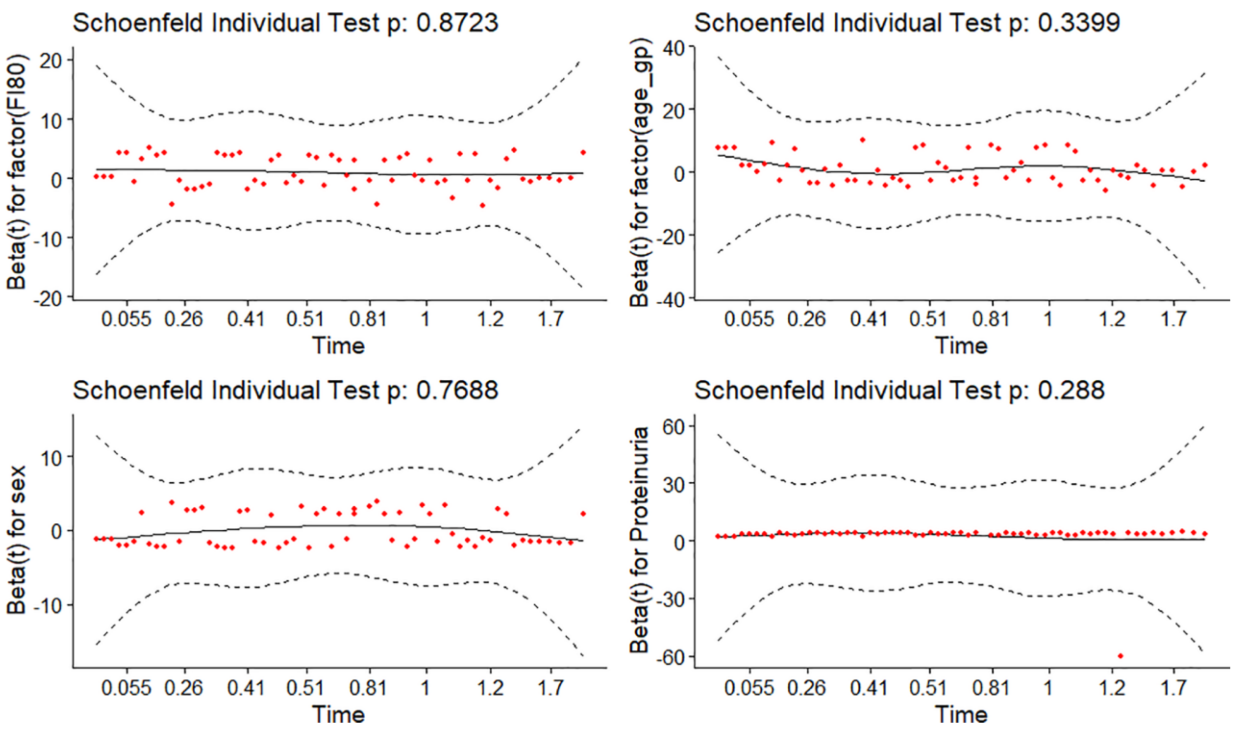

Supplementary Figure 1. The proportional-hazards assumption with the Schoenfeld residuals test in the association of FI80 with clinical outcomes.
